# Sustaining innovation in the health care workforce: A case study of community nurse consultant posts in England

**DOI:** 10.1186/1472-6963-11-200

**Published:** 2011-08-20

**Authors:** Vari M Drennan, Claire Goodman

**Affiliations:** 1 Faculty of Health & Social Care Sciences, Kingston University and St. George's University of London, Cranmer Terrace, London SW17 0RE, UK; 2 Centre for Research in Primary and Community Care, University of Hertfordshire, College Lane, Hatfield, Herts AL10 9AB, UK

## Abstract

**Background:**

Recruiting, retaining and meeting increasing demand for experienced, qualified nurses is an issue of concern for all health care systems. The UK has been creating clinical career structures for nurses that include innovative posts known as nurse consultants. While the numbers overall appear to have grown over the last eleven years, there is evidence that in some specialities and regions the numbers are decreasing. This paper considers the factors that sustain or curtail workforce innovations through the case example of a cohort of nurse consultants established in one community health service in England.

**Methods:**

A mixed method case study evaluation was undertaken over three years, using interviews, observations, documentary analysis and questionnaires. The final element of data collection was obtained three years later. Data was anonymised, analysed using a framework method and then integrated using a narrative synthesis.

**Results:**

Ten nurse consultant posts were created over a period of two years (2002-2004). Within two years only five posts remained and within five years (2009) only two part time posts, with the original appointees, remained. When the nurse consultants left their posts, these were not replaced. In exploring the interaction between the innovation (the nurse consultant posts), the adoptees (the senior staff in the organisation) and the context (the immediate service colleagues, the service organisation and commissioners as well as the broader NHS policy context) three key factors were identified as influential in the demise of the posts. These were: a) the extent to which there was support for individual nurses rather than the post, b) the extent to which there was an unambiguous and uncontested clinical service requirement for a nurse consultant and c) the extent to which finances for the post were judged as being used to best effect in a service setting.

**Conclusions:**

This case study example demonstrates how tantalisingly close the nurse consultant initiative came to achieving a clinical career pathway for nurses in community services but more work was required to normalise clinician and managers' beliefs in the relevance and need for the role. Those looking to embed these types of nursing workforce innovations should pay due regard to these issues.

## Background

Recruiting, retaining and meeting increasing demand for experienced, qualified nurses is an issue of concern not just for individual organisations but for government agencies throughout the world [1,2]. Human resource analysts offer a wide range of solutions from the economic e.g. attractive pay and benefits, to the wider social advantages e.g. workplace nurseries [3,4]. Many commentators have argued that the career structure for nursing in most countries takes the experienced nurses away from direct patient contact and into management or education spheres [5]. The UK like other countries [6,7] has been exploring ways of creating clinical career structures for nurses that retain experienced nurses in clinical practice rather than move to management or teaching roles or leave. One example of this has been the creation of a senior nurse role within the National Health Service (NHS) called a 'nurse consultant' [8]. This new role, specifically designed to offer experienced clinicians an alternative to education and management options with equivalent levels of remuneration, was launched in 1998 by the Prime Minister. Since then the numbers in England have gradually increased [9] but a more detailed analysis demonstrates fluctuations and even decline in numbers in some specialities (see below). While there is much written about the introduction of innovation [10], such as new roles in health care, there is little that considers the factors that sustain innovation [11]. This paper considers the factors that sustain or curtail workforce innovations through the case example of a cohort of nurse consultants established in one community health service in England.

### Nurse Consultant Posts in England

Nurse, midwifery and health visitor consultant posts were introduced in the UK under a government commitment to improve the quality of health services and provide a clinical career path for senior and experienced nurses [8]. Following a national consultation exercise the objectives of post were stated in implementation guidance:

• "To help provide better outcomes for patients by improving services and quality,

• "To strengthen leadership,

• "To provide a new career opportunity to help retain experienced and expert nurses, midwives and health visitors in practice." 12 [paragraph 5].

While the role name has been used in other countries such as Australia [13] and the USA [14] and is currently being piloted in some Hospital systems such as Hong Kong [15], the UK has been unusual in specifying at a national level the core functions of these posts:

• An expert practice function (with fifty per cent of time in clinical practice);

• A professional leadership and consultancy function;

• An education, training and development function; and

• A practice and service development, research and evaluation function. 12 [paragraph 6].

Nurse consultants (used in this paper to include midwife and health visitor consultants) were expected to have Masters level education or equivalent [12] but there was no explanation as to how these posts differed from pre-existing senior clinical roles such as clinical nurse specialists. Each of the four countries within the UK adopted slightly different approaches to the introduction of nurse consultants' roles with England being the most proactive. The first 230 nurse consultant posts were authorised by the Department of Health (England) in 2000 [12]. After 2001 this central authorisation for the posts was not required. The past ten years have seen numerous personal accounts [see for example 16], descriptions of the activities undertaken [17,18] and studies of perceptions of the achievements of the roles [19-21]. Early and more recent studies of the establishment of the new nurse consultant posts suggested that factors such as supportive workplaces, realistic workloads and continuing education were important for the consultant nurses to perceived they were effective in their roles [22-25]. There have been no studies of effectiveness or cost effectiveness [23].

**Figure 1:**
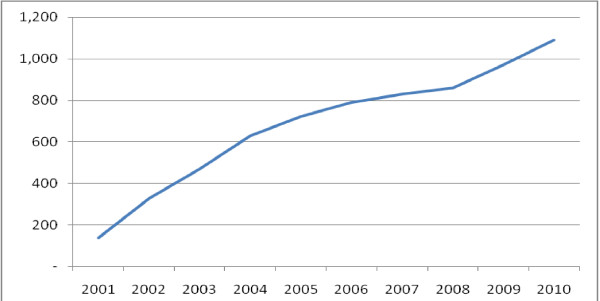
**The number of nurse consultants (head count) in England 2001 -2010**. Source: NHS Information Centre. NHS HCHS: Nursing, Midwifery & Health Visiting staff and support staff by type 2000-2010 Table 2a. 2011.

The government aimed to have 1,000 of these posts established in England by 2004 [26]. By 2008 there were 851 nurse consultants in post and this had risen to 1,091 by 2010 [9] accounting for 1,024 full time equivalent posts and 0.3% of the qualified nurses employed in the National Health Service (NHS) England. The overall number of nurse consultant posts has increased since 2001 with the rate of growth slowing in 2004 and then a sharper increase in 2009 (Figure 1). This overall picture of growth however masks both variation in regional distribution and in specialities and also decline some of these.

**Figure 2:**
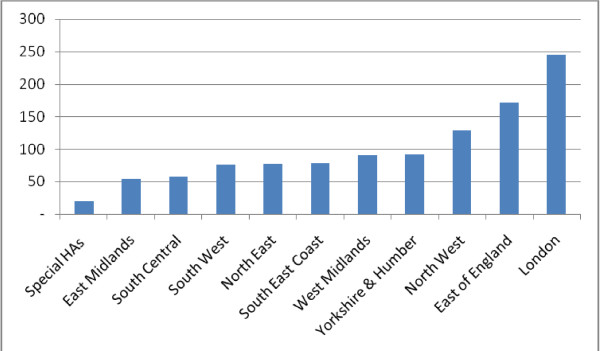
**The distribution of nurse consultant posts between Health Authorities 2010**. Source: NHS Information Centre. 2011 NHS HCHS: Nursing, Midwifery & Health Visiting staff and support staff by type 2000-2010 Table 2a.

The number of these posts varies greatly between the NHS regions, known as Strategic Health Authority (SHA) areas of England: the lowest number in the East Midlands and the highest in the London SHA area (Figure 2). The size of population served does not explain the variation in numbers. For example, the North West SHA serves 7 million people but has about half the number of nurse consultants in post of the London SHA serving seven and half million people [27]. The North East SHA serves the smallest population of two and half million but has equal number of nurse consultants to the South West SHA, which has twice its population [27].

**Figure 3:**
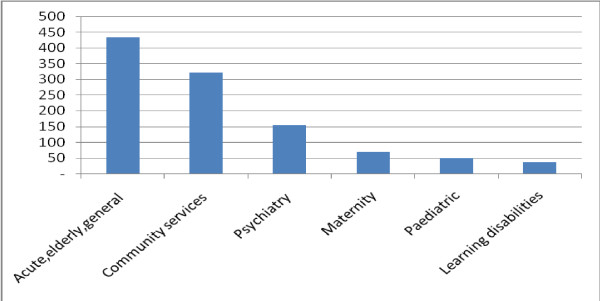
**The distribution of nurse consultant posts (head count) between service areas 2010**. Source: NHS Information Centre. NHS HCHS: Nursing, Midwifery & Health Visiting staff and support staff by type 2000-2010 Table 8. 2011.

**Figure 4:**
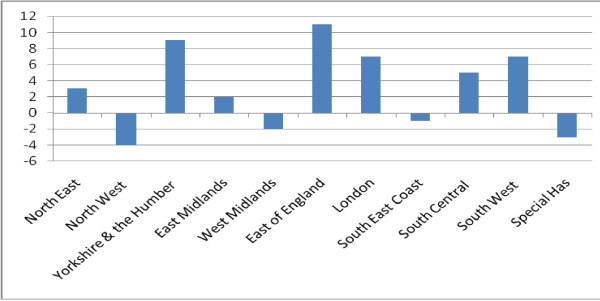
**Change in full time equivalent community services nurse consultant posts by strategic health authority between 2006 and 2008**. Source: NHS Information Centre. NHS HCHS: Nursing, Midwifery & Health Visiting staff and support staff by type 1998-2008 Table 8. 2007,2008 and 2009.

Variety is also demonstrated in the frequency of the posts in different clinical service areas. The greatest numbers are found in acute, older people and general hospital services, followed by community services and then psychiatric services (Figure 3). More detailed examination shows that while overall trends appear to indicate growth, there are SHA areas where there has been a decline in the number of posts. Eight strategic health authorities reported increased numbers between 2008 and 2010 [9], two reported the same number and one reported a decline. This variation is reflected in clinical service areas. For example between 2006 and 2008 there was a decrease in the number of community nurse consultant posts in three strategic health authorities (Figure 4). Other evidence confirms this trend for certain specialist areas, a survey of Directors of Nursing in health care organisations in England found that they anticipated a decline in numbers of nurse consultants in paediatrics although the report offers no explanation [28].

### Theoretical Frameworks of Innovation in Health Care

There is a long history of nursing in the UK of creating new roles to address demand for specialist nursing expertise which have titles to reflect the additional expertise and training of senior clinicians e.g. clinical nurse specialists, nurse practitioners, community matrons [29]. However, these roles have often developed in an *ad hoc *manner, with time limited funding, tied to local settings with little national consensus about their remit, range of responsibilities or even titles [30]. There has been an ongoing failure to embed senior clinical nurse roles in ways that ensure they are a recognised and predictable part of the national workforce. From an organisational point of view, embedding and sustaining an innovation until it is part of the delivery system is a complex, poorly understood process [31]. Greenhalgh et al [11] and others suggest that widespread uptake depends on the interaction between features of the innovation, the adopter(s), and the context. Key features of the innovation for adoption include: that it has a perceived relative advantage, that it is compatible with perceived needs, values and norms, that it has low complexity, that it is amenable to being tested out on a limited basis, that the benefits are observable, and that the potential for reinvention or adaption to local circumstances (adapted from Greenhalgh et al [11]). Greenhalgh et al [11] argue that an innovation is more likely to be assimilated when it fits with the organization's existing values, norms, and ways of working and supporters of the innovation outnumber and are more strategically placed than its opponents. This paper takes the example of nurse consultants as an innovation in the nursing workforce that had national and professional endorsement and appeared to fit with a health care organisation's values and priorities for patient care. It reports on one aspect of a case study of a cohort of nurse consultants recruited in one community health service organisation. The overall case study addressed questions of the impact of nurse consultants on patient care and the effect of a cohort of nurse consultants in an organisation. This paper reports on the third research question: to what extent did this workforce innovation became assimilated over time and what factors influenced that?

## Methods

A mixed method case study evaluation [32] was undertaken that drew on methods of participant enquiry. The data was collected over three years (2003-2006) through:

• Semi-structured individual and group interviews with nurse consultants repeated over time, and at exit from employment where feasible,

• Questionnaires to service colleagues nominated by individual nurse consultants,

• Observation of nurse consultants in clinical areas and in service meetings,

• Organisational and service documents,

• Semi-structured interviews with stakeholders, including medical consultants, nurses, allied health professionals, middle and executive managers, service commissioners.

The authors were responsible for all elements of data collection. The individual semi-structured interviews with the nurse consultants were framed around topics which explored: a) their initial motivation in applying for the post, b) their role and current and planned work activities, c) barriers and facilitators in achieving their role aims and d) reflections on their experience as a nurse consultant on leaving. The interviews were conducted every 4-6 months with each nurse consultant and also at points of critical events such as the organisation splitting into two. Interviews were taped, with permission, transcribed and then deleted. Eight group interviews with the nurse consultants were held to explore similarities and differences in the nurse consultant activities, evidence of impact in the services and emerging issues for the nurse consultant role. These group interviews were also used to present, discuss and validate emerging findings from the case study evaluation. These group interviews were taped, with permission, transcribed and then deleted. The interview data was thematically analysed by the two researchers using constant comparative methods [33] at regular intervals through the study period. Emerging findings were then tested in the group interviews and informed the next set of interviews. Two nurse consultants agreed to questionnaires to be sent to service colleagues whom they nominated. These questionnaires included questions of interest within the study with adapted elements from a 360° feedback questionnaire, in use locally in the NHS at the time and unpublished, which explored the perceptions of service colleagues (both senior, junior and at the same level) as to leadership style and abilities of the individual. The questions explored areas such as clinical credibility, strategic vision and communication. Space was left for free text and additional comments (Additional file 1). Thirty questionnaires were sent to named individuals by the researchers and returned anonymously but indicating post type. The collated, individual report was returned to the nurse consultant and discussed in an interview. Two nurse consultants agreed to observation in clinical activities and with permissions of individual patients and colleagues the researchers observed the nurse consultants in clinical activities at eight sessions (ranging in time from one to three hours). The observations focused on the range and types of activities of the nurse consultant with patients and staff. Field notes were made and checked with the nurse consultant for validity.

The researchers observed forty service meetings in which one or more nurse consultants were present. The type of meeting ranged from individual service review to organisation wide nursing policy and development. The observations focused on the range and types of activities of the nurse consultants within these meetings and the interactions with other staff groups. Field notes were made, synthesised and discussed as part of the group interviews. Thirty five semi-structured interviews were undertaken with stakeholders, as listed above, in the second and third year. The topics explored included views on the contribution of the nurse consultants to the service and organisation and factors that supported or hindered the contribution. For those in senior positions aspects of the establishment of the posts were explored. Sixty public and internal organisational and service documents, e.g. service plans and reviews were obtained during the period. These were analysed for evidence concerning the history, infrastructure, support, achievements and any issues related to the nurse consultant posts.

The final element of data collection regarding the continued employment and role of the nurse consultants was obtained from interviews with senior managers in 2008 and 2009.

Each element of the data collection was analysed separately and at the point of the collection as described above. Data that pertained to this question were identified from each element and analysed using a framework method [34], whose categories evolved in the iterative analysis over time.

The study received a favourable reviewed from a local National Health Service (NHS) Research Ethics Committee.

## Results

The findings are presented in the following order: firstly, information on the case study site with details of the nurse consultant posts, their activities and the reduction in numbers, and secondly, a thematic analysis of the factors which influenced that decline.

### The case study site

The case study site was a primary care organisation (PCO) providing ambulatory, domiciliary and some inpatient services for 300,000 residents in an English city. It employed over 1,000 staff. It also provided clinical training placements for medical, nursing and allied health professions students. The PCO was judged by the external regulatory processes to provide good quality services, have good financial management and its achievements in innovative human resource policies were recognised by the Department of Health. A NHS administrative reorganisation, one year after the study commenced, resulted in the PCO subsequently dividing into two new independent organisations.

### The nurse consultant posts and activities

The PCO established ten nurse consultant posts over a period of two years (from 2002-2004). The posts were in: palliative care, older people, tissue viability, tuberculosis, sexual health, children, long term conditions, continence, primary care and public health. The job descriptions emphasised four areas of duties and responsibilities: expert clinical practice, education and continuing professional development, research & audit and leadership and consultancy. All the job descriptions stated that the purpose of the role was to develop both services in the named speciality and also excellence in the quality of the nursing. The scope and extent of the speciality varied between the posts. For example the nurse consultant in sexual health was based within a multi-disciplinary, ambulatory single clinic setting while the long term conditions post was within the PCO community nursing services but expected to work with over 30 general practices in the area. Five of the nurse consultants were already working in the organisation at appointment. Three were recruited from outside the organisation. The post for tuberculosis services was never advertised, suggesting some ambivalence in the service to these posts, and for the post for continence was advertised twice but the post was not filled. Within two years of the ten nurse consultant posts being created only five remained and within five years (2009) only two part time posts, with the original appointees, remained. The details of each post are listed below.

• Post 1. Established in 2002 and the post holder left after 6 months. The nurse consultant post was not replaced.

• Post 2, Permission given to establish the post but not progressed. The service remained staffed by clinical nurse specialists.

• Post 3. The post was advertised, interviews were held but no appointment was made. The service remained staffed by clinical nurse specialists.

• Post 4. Established in 2002 and the initial post holder remained in post. The post holder was appointed part time to undertake another senior nursing role across the organisation in 2008. The hours given to the senior organisational role were not replaced by another nurse consultant,

• Post 5.Established in 2003. The initial post holder left in 2009. The nurse consultant post was not replaced.

• Post 6. Established in 2003. The post holder moved to a senior position in the service in 2005. The nurse consultant post was not replaced.

• Post 7. Established in 2003. The post holder moved to a senior position in the organisation in 2005. The nurse consultant post was not replaced.

• Post 8. Established in 2003. The post holder left in 2004. The nurse consultant post was not replaced.

• Post 9 Established in 2002. The post holder left in 2009. The nurse consultant post was not replaced.

• Post 10. Established in 2004 and the initial post holder remained in post. The post holder was appointed to undertake another senior role part time at a national level in 2009. The part-time hours given to the national role were not replaced by another nurse consultant.

When the individuals left their posts, the posts were not replaced. The nurse consultants left to either join other organisations in more senior management positions or to leave the NHS. None left their posts for other nurse consultant posts.

All the nurse consultants undertook activities in the four key areas of their job descriptions. The extent to which they engaged in all of them reflected something of their service context and length of time in the organisation. All but one undertook direct patient clinical activity. While most commenced their posts with the intention of having 50% of their time in clinical activity only one of them achieved and sustained this level. The direct clinical activity ranged from being part of the medical consultant on-call rota, conducting their own clinics (i.e. nurse led clinics) to being the key worker/case manager for a small group of patients. Indirect clinical activity undertaken by the nurse consultants included clinical supervision of other nurses, providing specialist advice on specific patients or families to nurses or other professional staff, and undertaking review of services from a clinical or professional perspective.

Two nurse consultants became the clinical lead for their multi-disciplinary team, including medical services. One of these was for a temporary period of some months while the medical consultant was on long term sickness leave, the other was appointed following a medical consultant stepping down.

All of the nurse consultants were participants, often in leading roles, in service and organisation wide, quality assurance and development committees. Three nurse consultants were part of regional and national networks formed to improve the quality of care to specific patient groups through benchmarking, setting clinical standards, describing staff competencies and providing training. Most were involved, sometimes leading, in audit, clinical review and service evaluation activities at various points

Half of them undertook education and teaching activities in work based sessions and University programmes. Five undertook some research activities at some time over the period, mainly through the pursuit of higher degrees. Two nurse consultants gained masters degrees, one gained a PhD by research and one gained entry to the national Public Health Register. Four nurse consultants had articles published, including research publications, descriptions of audits and reflections on the experience of becoming a nurse consultant. Two nurse consultants had their activities reported in the national nursing press and one was publicly named and thanked by a patient in a feature article in a national newspaper. One nurse consultant was recognised in the UK Honour System.

Five of the nurse consultant posts were established with the intention that they were direct line managers for other nurses. A further two nurse consultants took on the operational management of staff and services for some months at points when the service manager was absent through vacancy or long term sickness absence. Two nurse consultants were part of the service management team engaged in regular negotiations and review with the commissioners of their services. Two nurse consultants became members of service commissioning review mechanisms.

The thematic analysis identified the following issues influencing the extent to which the nurse consultant posts were embedded and sustained in the organisation: support for individual nurses rather than the post of nurse consultant, the contested nature of the nurse consultant roles and finally the resource implications of new roles.

### Support for the person not the post

The importance of the personality and competence of the initial post holder in the new nurse consultant role was evident early on in the evaluation: for example in one speciality the medical consultants reported that if the current nurse consultant left it was not certain that they would advertise the post to be exactly the same. As she said:

"It is not automatic that we'd continue. It is so much about Z [name]. Someone else couldn't fulfil what Z is doing exactly. So we have not had discussions about the other nurse consultants or about Z's role". Medical consultant 1

Senior staff observed that some individuals had greatly over-performed in their previous specialist nurse roles so that they were able to hit the ground running when they were appointed to nurse consultant posts,

"Y [name of post holder] was straining at the leash but didn't have the teeth, so much of the development of the nurse consultant post was already taking place, but it didn't have the mandate the post bought. Y was acting as a nurse consultant (prior to the post being established) and we could capitalise on this". Medical consultant 3

Individual factors were important for the development of the posts. Senior managers and medical consultants linked specific successes of the posts with the particular attributes of the post holder.

"It is more X [name of post holder] than the role-X was doing some of it before. The nurse consultant role confers recognition and status. I'm not sure we'd have had the impetus to create the post if we hadn't had X. X is a champion for nurses in the multi-disciplinary team, and that is all to the good". Medical consultant 6

While the Director of Nursing and some of the nurse consultants highlighted early on the need to develop other nurses to be ready to take new nurse consultant posts, it was not evident that any work was progressed in this area. Succession planning for nurse consultants was a topic raised briefly on one occasion in the nurse consultant group interviews but not pursued. Therapy and medical consultants in interviews contrasted this with the specified routes of training and examinations provided by their professional bodies to the absence of such for nurse consultants in the UK.

### Supported, Contested and Ambiguous Roles

The motivation for establishing the posts were described variously by the senior executive team members but included: a willingness to test a workforce innovation that would be an explicit change agent for nursing and concerns as to how to retain experienced nurses in clinical leadership roles. For those senior managers with nursing backgrounds there was also an aspiration for the profession of nursing as indicated here:

"It started from a dream-a belief that we need clinical leadership at the highest level, integrated with teaching and research. It is about senior practice, linked into service provision. It can be the highest point of a nursing career."Executive Team member 3

While the establishment of the nurse consultant posts had support at the highest level in the organisation there was evidence of greater uncertainty from other groups within the organisation. This was expressed initially by doctors, clinical nurse specialists and clinical service managers, in the main, centred around concerns of encroachment of work roles and spheres of influence. One medical consultant who was generally very enthusiastic about nurse consultants expressed this concern:

"If nurse consultants see themselves as leading lights in the nursing world, that's great: if the nurse consultants see themselves in the medical camp that is not so helpful". Medical consultant 5

These types of role boundary concerns were shared by some of the nurses particularly clinical nurse specialists. When the nurse consultant posts were created, some clinical nurse specialists lost lead responsibilities for activities such as nursing practice policy development and audit to the nurse consultant.

*"The scope of my role, for which I was awarded 3 discretionary points *[on the salary scale] *has been substantially reduced because of this *[nurse consultant] *post". Clinical nurse specialist 3*

In some services this caused initial resentment which disappeared over time; in others the clinical nurse specialists continued to dispute the impact of this new role on their potential contribution and job satisfaction.

The clinical service managers, the majority of whom came from nursing backgrounds, could initially see more potential for contested ground.

"But that's what I do now, provide clinical leadership to nurses-how is this going to work with me and a nurse consultant? Will I do less? Who will be making decisions about the priorities of the nursing service-me or the nurse consultant? May be I should be called the nurse consultant". Clinical service manager 4

While the senior managers and lead medical consultants were emphatic early on that the nurse consultant role was not to be a management role, the boundary between clinical leadership, service development and management was not clear, either as a concept or in practice. From early on, nurse consultants stepped in to clinical service manager roles when there were gaps caused by illness or re-organisations. As time went by, managers and nurse consultants both shaped the role towards assuming management responsibilities. Overall there seemed to be an inclination to shape the role into a known model of professional leadership:

"The nurse consultant post is slightly anomalous, as it does not fit naturally into the management structure. V [name of post holder] is not a manager and doesn't control other staff. I can understand the reluctance to include management in the post, but V would be ideal to manage some of the specialist nurses. It would be analogous to medical consultant posts, which do include clinical duties and management". Senior manager 5

The nurse consultant roles and work developed most smoothly when a) their sphere of clinical activity did not overlap with others and b) when both service managers and medical colleagues agreed with the direction of their work of the nurse consultant.

"Our nurse consultant has worked well. What reassured us about our post was that what W does is not a role that others were concentrating on". Medical consultant 3

Where there was a lack of clarity and agreement in the sphere of the work and the responsibilities problems and conflicts arose. One post had very early on not worked out as well as expected in its original form: concerns were expressed by a medical consultant and echoed by a manager who considered that the post holder and the service had different perspectives on what it could achieve.

"It is not clear how they interface with medical consultants. No-one was clear what the nurse consultant was there to do clinically". Medical consultant 8

From the individual nurse consultants perspective their service context was significant in decisions to remain or not. Opportunities to innovate within and improve their services were important to the individual nurse consultants, as was the sense of organisational support. Nurse consultants who left relatively quickly from their posts were in services where other senior staff or commissioners contested their desired work activities and proposed developments. Aspirations of individual nurse consultants to provide nurse led specialist clinics in primary care were often frustrated by lack of support both from general practitioners and by hospital consultants arguing that this was not best use of resources.

### The resource implications of new roles

While some managers discussed tangential consequences for the organisation of creating these posts such as improving recruitment and retention of nurses, in the main the aspiration was that the nurse consultants would contribute to both quality improvements and cost reductions.

"Nurse consultants will lead to new initiatives, making the most of high-tech and low-tech opportunities, and making better use of scarce resources". Executive team member 5

However, the resource implications of the nurse consultant posts were an area of uncertainty within the organisation;

*"We have taken money out from various sources *[to fund the nurse consultant post], *but we don't yet know if nurse consultants save or cost money. It is more about raising standards of care". Senior manager 2*

While one senior manager described it as "*an act of faith*" in financing the posts with the hope that they would assist in reducing costs, a medical consultant pointed out that if they fulfilled the aspiration to develop services they might actually increase costs. Much more of the resource implication debate articulated by senior staff in the organisation centred on the degree to which nurse consultants would substitute for doctors and how to judge the cost consequences. The difficulty the organisation had in doing this is expressed in this exemplar quote:

*"They are cheaper than medical consultants and that is part of the push *[to employ nurse consultants]. *90% of our work can be done by nurses working to guidelines. If nurses are used for routine work they are cheaper than doctors. But it is hard to say exactly, as most doctors' work is done by junior grades and not by consultants. And nurses have longer appointment times. If we had to level that out, what would the costs be?" Medical consultant 4*

Senior managers were cognisant of the challenge in evaluating both financial and service quality consequences of these posts in specific service contexts.

"There are financial implications. The posts cost money. But we are not sure if it saves money and that's the question we need to be able to answer. Is it a more effective way of managing patients? It is complicated and may be a similar dilemma to NHS Direct [a national telephone helpline] i.e. at worst it can be another built-on layer. It may improve quality or nurse consultants may duplicate what doctors already do and the patients may have to see the doctor anyway". Senior manager 4

Throughout the period of the study, the primary care organisations were required by their commissioners to identify cash releasing efficiency savings for reinvestment elsewhere. In such an environment, every service was reviewed internally and all posts that became vacant, including those of the nurse consultants, was scrutinised as to whether the financial resource was being used to best effect. Within individual service budgets as each nurse consultant left their post, the monies were re-deployed to other posts and the post deleted.

## Discussion

This case study found that within two years of the ten nurse consultant posts being created in a primary care organisation only five remained and within five years (2009) only two part time posts, with the original appointees, remained. As a workforce innovation, the conclusion must be, that in this community services setting the nurse consultant roles were not successfully assimilated into the health care system.

The study explored the interaction between the innovation (the nurse consultant posts), the adoptees (the senior staff in the organisation) and the context (the immediate service colleagues, the service organisation and commissioners as well as the broader NHS policy context) and identified key factors which were influential in the demise of the posts. These factors were: a) the extent to which there was support for individual nurses rather than the post, b) the extent to which there was an unambiguous and uncontested clinical service requirement for a nurse consultant and c) the extent to which finances for the post were judged as being used to best effect in a service setting.

This case study demonstrates that the nurse consultant roles in this setting did not meet many of Greenhalgh et al's [11] features of the innovation required for successful adoption. Pettigrew et al [35] in their study of organisational innovation in the NHS, identified that the receptiveness or not of local contexts was critical in the success or otherwise of implementing and sustaining change. May et al [31] have argued that there are four groups of factors in their normalisation model for embedding innovations. These groups of factors are: interactional workability i.e. the congruence of the innovation with existing people and practices, relational integration i.e. the extent to which the innovation integrates with the knowledge already used in that arena, skill set integration i.e. the extent to which the innovation fits with efficient division of labour as understood by those in that arena and contextual integration i.e. the relationship of the innovation to the wider organisation. In this case study there is evidence that these posts had difficulties in some or all of the four groups of factors. These posts did not survive as their remit was contested, engagement with influential players was variable, the role were not understood by other practitioners, clinicians and the clinical managers' beliefs about how the workforce should be organised and what effective patient and multi-disciplinary care should look like.

Recent [24,36] and earlier literature investigating the nurse consultant innovation has focused on the individual nurses rather than the organisational context although early evaluations noted the role confusion and ambiguity [19,21]. This study provides insights over time from the organisational perspective of nurse consultants as a workforce innovation that has not been provided before.

This case study was of a small cohort of nurse consultants in two primary care organisations in one English city. The study has limitations such as being undertaken in one (then two) primary care organisation although the organisation was similar to many other in cities in the UK. Those willing to be interviewed or provide views may only have been those with strongly held negative or positive views. However, this study by using a variety of sources, from a wide range of stakeholders and over time has gone someway to securing a full picture of the nurse consultants as a workforce innovation in community service settings.

The creation of the nurse consultant role by central government was echoed in the creation of two other nursing roles "the modern matron" [26] and the "community matron" [37]. While the modern matron posts grew and flourished with a defined, uncontested clinical management responsibility [38] the implementation of the community matron role has been much slower and contested [39]. Any innovation in health care workforce sits within a wider landscape and history of an interactive, contested system of changing professional and occupational boundaries [40]. This case study suggests that the contextual factors are critical in sustaining innovation in nursing workforce. This is particularly true in community and primary care settings, where the developments in nursing and the creation of new roles are driven by organisational needs to extend and substitute for medical provision, supplement existing nursing services and work across secondary and primary care [29]. Even a cohort of nurse consultants who were able to articulate collectively the importance of senior clinical roles in the organisation, were unable to delineate discrete areas of work that everyone recognised as nurse consultant work. Ultimately, this meant the role was always subject to change and redefinition and tied to the achievements of individuals as opposed to its ability to deliver on key patient and service outcomes.

These findings are specific to community settings. It is not known if or how nurse consultant posts in secondary care have over time become an integral part of service delivery. This requires further investigation. NHS workforce census data up until September 2010 shows continued growth in the number of nurse consultants in post. It is not possible to discriminate from the published figures whether the growth is in organisations who have not previously had these posts or whether the growth is occurring in new specialities in the same organisations. As in other health systems following the fiscal crisis, the NHS in England is required to increase productivity and reduce costs: it remains to be seen the impact of this environment on the numbers of nurse consultant posts.

## Conclusions

The findings of this study demonstrate that recognition of the need to retain experienced nurses as providers of patient care is not sufficient to embed striated occupational roles such as nurse consultant within primary care and community organisations. Nurse consultants were new roles that were superimposed on existing services and as a consequence had to repeatedly negotiate and justify their role and purpose. This was easier to achieve when nurse consultants addressed service priorities that no other professional group believed was their responsibility. Models of striated career advancement do not offer service providers the flexibility and responsiveness that current reactive approaches to the development of nurse clinicians do in the short term. However it is a wasteful approach that cannot be sustained. The challenge for all health care systems is to address increasing demands within available finances: clinical careers for nurses that ensure their expertise is retained, remunerated and shared with more junior staff is a key component of that. This case example demonstrates how tantalisingly close the nurse consultant initiative came in these settings to achieving that clinical career pathway but more work was required to normalise clinician and managers' beliefs in the relevance and need for the role. Those looking to embed these types of nursing workforce innovations should pay due regard to these issues.

## Competing interests

The authors declare that they have no competing interests.

## Authors' contributions

VMD and CG jointly conceived, designed and undertook the study. Both drafted and agreed the final manuscript.

## Authors Information

VMD and CG undertook the initial part of the study while leading the Primary Care Nursing Research Unit in the Department of Primary Care & Population Sciences, University College London, UK.

## Pre-publication history

The pre-publication history for this paper can be accessed here:

http://www.biomedcentral.com/1472-6963/11/200/prepub

## Supplementary Material

Additional file 1 **The 360° feedback questionnaire**. Click here for file
