# Supplementary material for: Sustaining innovation in the health care workforce: A case study of community nurse consultant posts in England
Source: BMC Health Serv Res. 2011 Aug 20;11:200. doi: 10.1186/1472-6963-11-200 (PMC3170598; doi:10.1186/1472-6963-11-200)
Supplement: Additional file 1 — The 360° feedback questionnaire. [file 1472-6963-11-200-S1.DOC]

Additional File 1 The 360 ^o^ Feedback Questionnaire

Text of Invitation letter

Dear colleague,

**Nurse Consultant in *[clinical title]* : 360 Degree Evaluation**

I am writing to ask you to help us provide a 360 degree evaluation of the nurse consultant in *[clinical title]* post undertaken by *[name].*

*[name]* has now been in post for a year and is currently evaluating progress so far and looking to plan for the coming eighteen months. It is very valuable to have the views of those who work most closely to reflect on the post to date and help inform future developments.

We are asking you to complete the attached questionnaire to help identify which parts of the role works well at present and those which could be developed differently. This feedback form is being sent out to a number of clinical colleagues and managers to {name].

Your individual response is anonymous and we ask you to return it in the pre-paid envelope within two weeks of receipt. Vari Drennan and Claire Goodman, who are undertaking the evaluation of the nurse consultants in the *[name of organisation]*, then collate the responses. A typed report of the feedback is then given to *[name]*. Individuals can not be identified. It is most helpful to anyone using these types of evaluations if comments can be framed in a constructive manner.

If you have any questions regarding this process you can ask [name] herself, or contact Vari or Claire at the above address. This evaluation has had a favourable review from the local NHS Ethics Committee.

Thank you for your time and help.

Your sincerely

**Nurse Consultant in [named clinical field]: 360 Degree Evaluation**

1. [name] has now been in post for a year as nurse consultant . Has the post developed in the way you anticipated? Please list ways in which it has developed and hasn’t developed as you anticipated.

2. Can you identify particular aspects of [name] nurse consultant work that have been supportive to your work /service/post?

3. Are there any aspects of your role/ service / practice that has changed directly because of this post?

4. Are there any aspects of the role you would like to see develop in the next eighteen months?

5. Given a single wish, what would be the one aspect that you would like to see this post make a difference to?

We ask you now to rate her competency from 1 to 5 in the different aspects associated with the role of nurse consultant. It is obviously helpful if you can be as honest and constructive as possible. There is also space for comments.

1 = needs development and 5 = excellent. Please circle the number that most reflects your views.

**1. Clinical Skills in [clinical area]**

1.1. Is able to demonstrate expert knowledge and nursing practice in [type of clinical activities specified]

| Needs development | ⇨ | ⇨ | ⇨ | Excellent |
| --- | --- | --- | --- | --- |
| 1 | 2 | 3 | 4 | 5 |

Comments

1.2. Able to use the available resources effectively in dealing with the emotional and interpersonal pressures of [clinical activities specified ]

| Needs development | ⇨ | ⇨ | ⇨ | Excellent |
| --- | --- | --- | --- | --- |
| 1 | 2 | 3 | 4 | 5 |

Comments

1.3 Is able to demonstrate expert knowledge and practice in [clinical care activities specified]

| Needs development | ⇨ | ⇨ | ⇨ | Excellent |
| --- | --- | --- | --- | --- |
| 1 | 2 | 3 | 4 | 5 |

Comments

1.4 Is able to demonstrate expert knowledge and practice in considering and addressing ethical issues {in the clinical care activities specified]

| Needs development | ⇨ | ⇨ | ⇨ | Excellent |
| --- | --- | --- | --- | --- |
| 1 | 2 | 3 | 4 | 5 |

# Comments

1.5 Able to makes sound judgements about clinical care and practice in situations which are complex and difficult

| Needs development | ⇨ | ⇨ | ⇨ | Excellent |
| --- | --- | --- | --- | --- |
| 1 | 2 | 3 | 4 | 5 |

# Comments

**2. Working in Partnership***

2.1. Establishes positive relationships easily and is skilful in interpersonal processes

| Needs development | ⇨ | ⇨ | ⇨ | Excellent |
| --- | --- | --- | --- | --- |
| 1 | 2 | 3 | 4 | 5 |

# Comments

2.2. Is able to work effectively in collaboration with others, including from different disciplines and organisations

| Needs development | ⇨ | ⇨ | ⇨ | Excellent |
| --- | --- | --- | --- | --- |
| 1 | 2 | 3 | 4 | 5 |

# Comments

2.3. Projects her self effectively and is confident without being arrogant

| Needs development | ⇨ | ⇨ | ⇨ | Excellent |
| --- | --- | --- | --- | --- |
| 1 | 2 | 3 | 4 | 5 |

Comments

2.4. Is effective in communicating and sharing information with others appropriately

| Needs development | ⇨ | ⇨ | ⇨ | Excellent |
| --- | --- | --- | --- | --- |
| 1 | 2 | 3 | 4 | 5 |

Comments

2.5. Is able to influence other people views and actions in a mature, non-abrasive manner

| Needs development | ⇨ | ⇨ | ⇨ | Excellent |
| --- | --- | --- | --- | --- |
| 1 | 2 | 3 | 4 | 5 |

Comments

2.6. Demonstrates a capacity to learn from others and from difficult experiences

| Needs development | ⇨ | ⇨ | ⇨ | Excellent |
| --- | --- | --- | --- | --- |
| 1 | 2 | 3 | 4 | 5 |

Comments

**3. Leadership***

3.1. Able to take responsibility and lead where appropriate without alienating others

| Needs development | ⇨ | ⇨ | ⇨ | Excellent |
| --- | --- | --- | --- | --- |
| 1 | 2 | 3 | 4 | 5 |

Comments

3.2. Encourages and motivates others when they are under pressure

| Needs development | ⇨ | ⇨ | ⇨ | Excellent |
| --- | --- | --- | --- | --- |
| 1 | 2 | 3 | 4 | 5 |

Comments

3.3. Able to think strategically and broadly about the whole organisation and how her speciality fits in

| Needs development | ⇨ | ⇨ | ⇨ | Excellent |
| --- | --- | --- | --- | --- |
| 1 | 2 | 3 | 4 | 5 |

Comments

3.4. Able to creatively envisage new ways of working and the steps to get there

| Needs development | ⇨ | ⇨ | ⇨ | Excellent |
| --- | --- | --- | --- | --- |
| 1 | 2 | 3 | 4 | 5 |

Comments

3.5. Proactively networks with particularly opinion leaders, both inside the organisation and outside

| Needs development | ⇨ | ⇨ | ⇨ | Excellent |
| --- | --- | --- | --- | --- |
| 1 | 2 | 3 | 4 | 5 |

Comments

3.6. Demonstrates a deep understanding of current policies and how they impact on the service and the role

| Needs development | ⇨ | ⇨ | ⇨ | Excellent |
| --- | --- | --- | --- | --- |
| 1 | 2 | 3 | 4 | 5 |

Comments

**Thank you for your help in this**

**Please feel free to add any other comments , including any views about this feedback form**

***These sections were adapted from a regional NHS 360^o^ feedback questionnaire in use at the time [33].**
